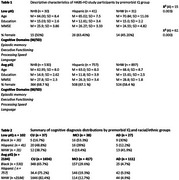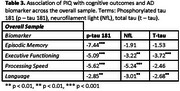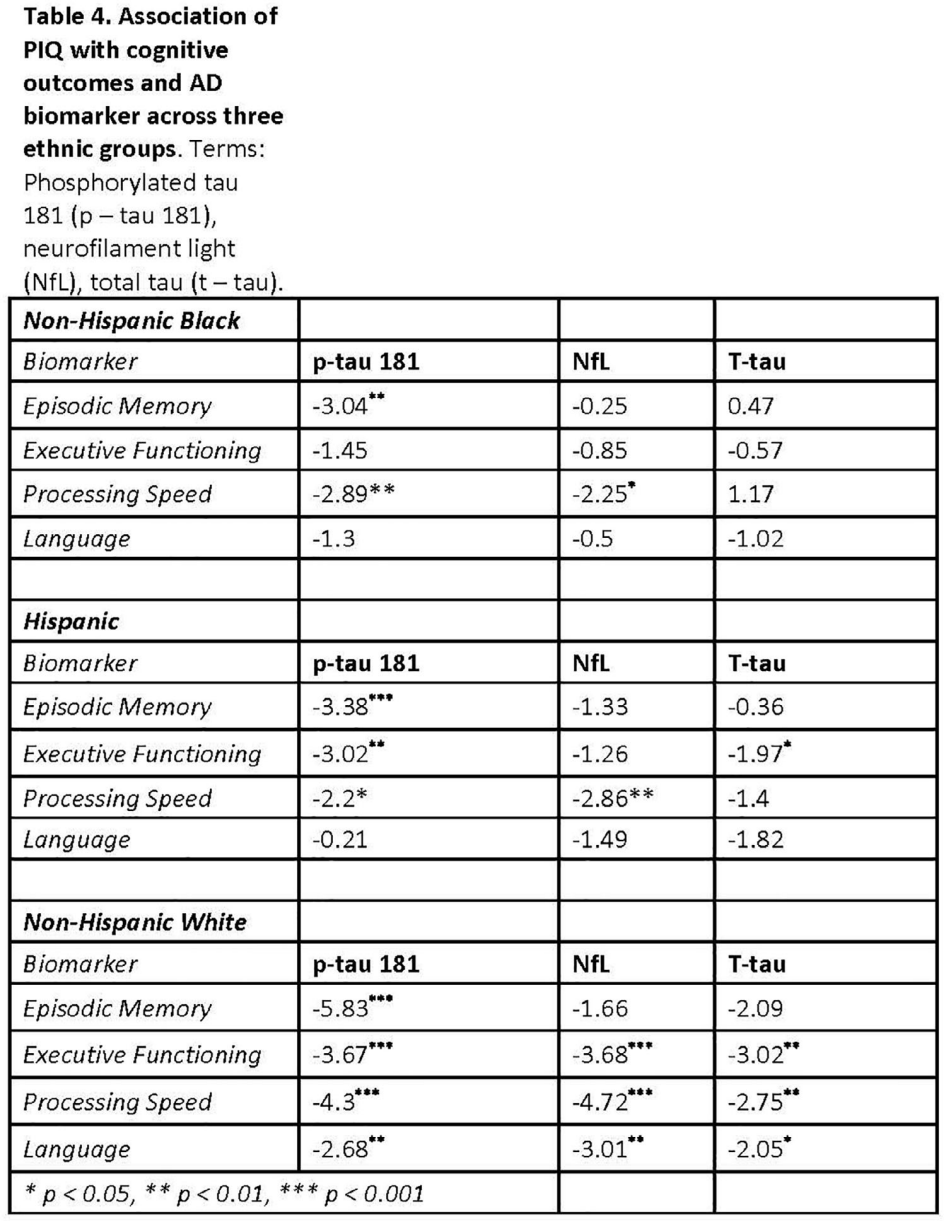# Association of Alzheimer's disease biomarkers with low premorbid intellectual functioning and cognition in a multiethnic community dwelling cohort: a cross‐sectional study of HABS – HD

**DOI:** 10.1002/alz70856_097218

**Published:** 2025-12-24

**Authors:** Lubnaa B Abdullah, Zhengyang Zhou, Mohammad Housini, James Hall, Sid E. O'Bryant

**Affiliations:** ^1^ University of North Texas Health Science Center, Fort Worth, TX, USA; ^2^ Texas College of Osteopathic Medicine, Fort Worth, TX, USA; ^3^ Institute for Translational Research, University of North Texas Health Science Center, Fort Worth, TX, USA

## Abstract

**Background:**

Individuals with intellectual/developmental disabilities (I/DD) have been underrepresented in Alzheimer's disease (AD) biomarker research, particularly those without Down syndrome (DS). This study examines the association between plasma AD biomarkers—phosphorylated tau 181 (*p*‐tau 181), total tau (T‐tau), and neurofilament light (NfL)—and cognitive outcomes based on premorbid intellectual ability (pIQ).

**Method:**

Participants were drawn from the Health & Aging Brain Study – Health Disparities (HABS‐HD), categorized by low (z ≤ ‐2.00) or average (z = 0.00 ± 1.00) pIQ based on word reading scores. Statistical analyses were conducted to evaluate whether biomarkers associated with cognitive domains of episodic memory, executive functioning, processing speed, and language by pIQ and ethnicity.

**Result:**

Among those with low pIQ, elevated plasma *p*‐tau 181 was associated with worse episodic memory. In stratified analyses, NfL, *p*‐tau 181, and t‐tau were associated with worse cognitive executive functioning among non‐Hispanic Whites (NHW) and Hispanics with low pIQ. All three biomarkers were associated with low pIQ and processing speed, with significant interactions based on ethnicity. Language was only associated with AD biomarkers for NHW's with low pIQ.

**Conclusion:**

These results highlight the importance of considering both premorbid IQ and ethnic background when examining the relationship between cognitive changes and AD biomarkers.